# Supplementary material for: The Systems Biology Research Tool: evolvable open-source software
Source: BMC Syst Biol. 2008 Jun 29;2:55. doi: 10.1186/1752-0509-2-55 (PMC2446383; doi:10.1186/1752-0509-2-55)
Supplement: Additional file 1 — SBRT Archive. An archive of the current version of the Systems Biology Research Tool. [file 1752-0509-2-55-S1.zip › sbrt-1.4.0/doc/developers_guide/api/sbrt/kernel/math/stat/comp/KendallTauB.html]

KendallTauB


|  |  |  |
| --- | --- | --- |
| **Overview** \| **Package** \| **Class** \| **Use** \| **Tree** \| **Deprecated** \| **Index** \| **Help** | |  |
| **PREV CLASS**   **NEXT CLASS** | **FRAMES**    **NO FRAMES**     **All Classes** |  |
| SUMMARY: NESTED \| FIELD \| CONSTR \| METHOD | DETAIL: FIELD \| CONSTR \| METHOD |  |


---


## sbrt.kernel.math.stat.comp Class KendallTauB<V>

```
java.lang.Object
  sbrt.kernel.math.stat.comp.KendallTau<V>
      sbrt.kernel.math.stat.comp.KendallTauB<V>
```

**Type Parameters:**: `V` - the variable type.

**All Implemented Interfaces:**: Correlator<V,TauResult>

---

``` public class KendallTauB<V> extends KendallTau<V> ```

This class is used to compute Kendall's Tau_B_ correlation
statistic.

**Author:**
:   This class was written and documented by Jeremiah Wright
    while in the Wagner lab.

---

**Constructor Summary**

`KendallTauB()`


| **Method Summary** | |
| --- | --- |
| `double` | `getTau(double[] xValues, double[] yValues)`             Computes and returns the correlation statistic for the provided values. |

**Methods inherited from class sbrt.kernel.math.stat.comp.KendallTau**

`corr, corr, corr, corr, getApproxZ, getApproxZ, getApproxZ, getS, getS, getS, getTau, getTau, getTieScore, getTieScore, getTieScore, getVarS, getVarS, getVarS, main`

**Methods inherited from class java.lang.Object**

`clone, equals, finalize, getClass, hashCode, notify, notifyAll, toString, wait, wait, wait`

**Constructor Detail**

### KendallTauB

```
public KendallTauB()
```


**Method Detail**

### getTau

```
public double getTau(double[] xValues,
                     double[] yValues)
```

:   Computes and returns the correlation statistic for the provided values.

    :   **Specified by:**: `getTau` in class `KendallTau<V>`
    :   **Parameters:**: `xValues` - an ordered array of values.: `yValues` - an ordered array of values. **Returns:**: the correlation statistic for the provided values.


---


|  |  |  |
| --- | --- | --- |
| **Overview** \| **Package** \| **Class** \| **Use** \| **Tree** \| **Deprecated** \| **Index** \| **Help** | |  |
| **PREV CLASS**   **NEXT CLASS** | **FRAMES**    **NO FRAMES**     **All Classes** |  |
| SUMMARY: NESTED \| FIELD \| CONSTR \| METHOD | DETAIL: FIELD \| CONSTR \| METHOD |  |


---
